# Prevalence of sarcopenia and its associated factors: the impact of muscle mass, gait speed, and handgrip strength reference values on reported frequencies

**DOI:** 10.6061/clinics/2019/e477

**Published:** 2019-04-02

**Authors:** Virgílio Garcia Moreira, Mariângela Perez, Roberto Alves Lourenço

**Affiliations:** I Laboratorio de Envelhecimento Humano - GeronLab, Faculdade de Ciencias Medicas, Universidade do Estado do Rio de Janeiro, Rio de Janeiro, RJ, BR; II Departamento de Medicina Interna, Faculdade de Ciencias Medicas, Universidade do Estado do Rio de Janeiro, Rio de Janeiro, RJ, BR

**Keywords:** Elderly, Gait Speed, Handgrip Strength, Prevalence, Sarcopenia

## Abstract

**OBJECTIVES:**

Sarcopenia is a common treatable geriatric condition. The aim of this study was to estimate the prevalence of sarcopenia and its associated factors in community-dwelling elderly living in Rio de Janeiro, Brazil, and to discuss the impact of different muscle mass, handgrip strength and gait speed cut-off values on the reported frequency of sarcopenia.

**METHODS:**

The health habits, functional capacity, and anthropometric measurements of 745 individuals aged ≥65 years from the Frailty in Brazilian Older People study were analyzed. The participants were classified into the following four groups: no sarcopenia, pre-sarcopenia, sarcopenia and severe sarcopenia. Univariate and multivariate regression analyses were performed. Muscle mass, handgrip strength and gait speed cut-off thresholds tailored to the sample and those proposed by the European Working Group on Sarcopenia in Older People were used to compare the prevalence rates of sarcopenia.

**RESULTS:**

Seventy-three percent of the participants were female, 61.9% were Caucasian, and the mean age was 76.6 years. The prevalence rates of sarcopenia were 10.8% and 18% using the sample-tailored and European consensus cut-off values, respectively. Sarcopenia was associated with advanced age (OR: 37.2; CI95%12.35-112.48), Caucasian race (OR: 1.89; CI 95% 1.02-3.52), single marital status (OR:6; CI95% 2.2-16.39), low income (OR:3.64; CI 95% 1.58-8.39), and the presence of comorbidities (OR:3.26; CI 95%1.28-8.3).

**CONCLUSION:**

In this study, the estimated prevalence of sarcopenia was similar to that reported in most studies after the tailored handgrip strength and gait speed cut-off values were adopted. A higher prevalence was observed when the cut-off values suggested by the European consensus were used. This indicates that the prevalence of sarcopenia must be estimated using population-specific reference values.

## INTRODUCTION

In Brazil, epidemiological and demographic changes mean that 650,000 elderly people appear in the population each year. Most have multiple chronic morbidities, and a considerable number have cognitive impairments and functional limitations 1. The increase in life expectancy has become a primary risk factor for morbidity and disability, displacing morbidity and mortality risks from younger to older groups. As a consequence, the entire society will become tasked with the care of a greater proportion of people with chronic diseases 2.

Included among the most important diseases affecting elderly people is sarcopenia. This is a geriatric condition ascribed to the ageing process; however, it can be treated and even cured 3. Muscle mass (MM) represents approximately 45% of the composition of the body. It is estimated that healthy individuals lose approximately 1% of their MM per year after the age of 30 years. For this reason, some authors consider the loss of MM to be the most dramatic and functionally significant change associated with ageing 4. Many published studies have indicated a drastic reduction in the quality of life and the prognosis of elderly people with sarcopenia compared to those without it 5. Patients with sarcopenia display disordered muscle tissue and metabolism. The cellular processes underlying the development and progression of sarcopenia cause negative outcomes by decreasing an individual's strength, mobility, and functional capacity 6.

Sarcopenia is directly associated with functional dependency, institutionalization, lengthier hospital stays, higher numbers of hospitalizations, higher mortality rates and higher healthcare-related costs. Nevertheless, sarcopenia does not have a well-established conceptual or operational definition 7. Its former operational definition was entirely based on MM 8,9. Recently, there has been evidence suggesting the need to expand this definition to include the measurement of muscle quality in addition to muscle quantity 10.

In the last few years, with the aim of consolidating the new evidence, several agencies and researchers have collaborated to develop criteria for the screening, diagnosis, and treatment of sarcopenia 11. To accomplish this, investigators proposed several tools and biomarkers for assessing sarcopenia. However, many of these protocols are dependent on the clinical context and on the cut-off values for items used to identify vulnerable individuals in each examined population 12. The use of various diagnostic criteria for sarcopenia has returned a range of prevalence rates spanning from 2 to 34% in epidemiological studies 13.

Early diagnosis and intervention are extremely important to arrest the disability cascade that accompanies a clinical condition with this potential prevalence and well-known negative outcomes. The aims of this study were to estimate the prevalence of sarcopenia based on MM, strength, and functionality and to analyze the relationships between sarcopenia and sociodemographic factors, functional ability, and health status in a sample of community-dwelling individuals living in Rio de Janeiro, Brazil. We also aimed to evaluate the impact of various MM, handgrip strength (HS) and gait speed (GS) cut-off values on the reported prevalence of sarcopenia.

## MATERIALS AND METHODS

This study was a cross-sectional analysis of the baseline data of the Frailty in Brazilian Older People study, Rio de Janeiro, Brazil (Fibra-RJ). The Fibra-RJ methodology is described elsewhere 14. Briefly, a longitudinal study evaluated 745 elderly (65 years and older) members of private health insurance plans living in northern Rio de Janeiro, Brazil. The study was conducted through home interviews, from January 2009 to January 2010 and assessed sociodemographic data, health habits, functional abilities, self-reported comorbidities, levels of physical activity, and cognition variables.

Sarcopenia was diagnosed and classified using the consensus diagnostic criteria proposed by the European Working Group on Sarcopenia in Older People (EWGSOP) 11. The four strata based on MM, HS and GS were as follows: pre-sarcopenia = low MM only, sarcopenia = low MM plus low HS or GS, severe sarcopenia = low MM plus low HS and GS, and normal = none of the aforementioned conditions.

To compare the impacts of different HS and GS cut-off values on sarcopenia incidence rates, two different approaches were used. First, the cut-off values were tailored to the sample. The individuals were considered weak or slow if they were in the first quintile in terms of MM, HS, and GS; second, the cut-off values were determined to be MM <5.45 kg/m^2^ and <7.26 kg/m^2^^8^ for women and men, respectively; HS <20 kgf and 30 kgf for women and men, respectively; and GS <0.8 m/s for both sexes 11.

A dynamometer (JAMAR-J00105) was used to assess HS, which was tested with three repetitions in the dominant hand. The average of these three measures, adjusted for sex and body mass index, was used to determine muscle strength. Functional performance was measured by GS using a chronometer to calculate the time required to walk 4.6 metres, adjusted for height and sex 15. In both approaches, MM was estimated using a formula proposed by Lee et al. 16 that evaluates total skeletal MM based on body weight, height, age, sex, and race. Individuals in the first quintile were considered to have low MM (Table 4) 15,17,18.

### Ethics approval

This study was funded by a grant from the National Research Council - Brazil (Conselho Nacional de Pesquisa - CNPq) and the Carlos Chagas Filho Foundation for Research Support of the State of Rio de Janeiro, Brazil (Fundação Carlos Chagas de Apoio à Pesquisa - FAPERJ). All subjects signed a consent form. The ethics committee of Hospital Universitário Pedro Ernesto approved the study.

### Statistical analysis

To describe the sample and to compare the prevalence rates of sarcopenia calculated by the various strategies, contingency tables were created and the Chi-square and Somer tests were used. For the multivariate regression, the sample was divided into the following two groups: sarcopenic and not sarcopenic; the pre-sarcopenic individuals were excluded from this analysis. Each variable with statistical significance in the univariate analysis (*p*<0.2) was included in the multivariate model. Severe sarcopenia and sarcopenia were considered to be one group because severe sarcopenia was underrepresented in the sample. The Hosmer-Lemeshow test was used to analyze the discriminatory ability of the model. All descriptive statistics were calculated assuming a 95% confidence interval (CI) and a significance level of 0.05. Analyses were performed using SPSS, version 23, IBM Software, 2015, Chicago.

## RESULTS

A brief report on the findings of the present study was presented in the poster session of the XXI World Congress of Gerontology and Geriatrics 19.

Of the 745 observed individuals (mean age 76.6±6.9 years), 70.3% were women, 61.9% were Caucasian, and 42.8% were married or living with a partner. Most had more than 6 years of education (73.6%) and an income greater than 2.1 times the minimum wage (83.1%). Of these individuals, 52.8% were dependent for instrumental activities of daily living (IADL), and 18.5% were dependent for basic activities of daily living. A large proportion of the sample (56.2%) reported their own health as good or very good, and 71.8% reported no falls during the previous year (Table 1). Hypertension and osteoarthritis were the most prevalent conditions (64.7% and 35.9%, respectively).

Table 2 displays the cut-off points tailored for this sample. The prevalence of sarcopenia according to the tailored cut-off values and the EWGSOP criteria are presented in Table 3. According to the consensus guidelines of the EWGSOP, the cut-off points for MM were 7.26 and 5.45 kg/m^2^ for men and women, respectively. The cut-off points for HS were 30 kgf for men and 20 kgf for women, and for GS, the cut-off values was 0.8 m/s for both sexes.

**Figure 1:**
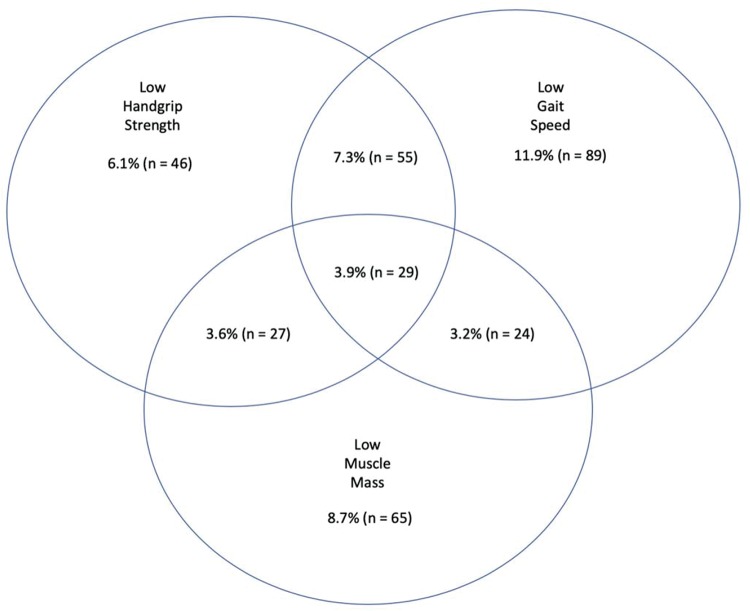
Venn diagram displaying the percentage of subjects [% (n)] who had low muscle mass and/or low handgrip strength and/or low gait speed amongst 745 individuals aged 65 years or older who are residents of the northern region of Rio de Janeiro, Brazil, 2010.

In the univariate regression model, race, age group, marital status, education level, income, comorbidities, and IADL were associated with sarcopenia. In the multivariate model, IADL did not maintain an association (Table 4). The distribution of MM, HS, and GS using the tailored cut-off values is shown in Figure 1.

## DISCUSSION

In this study, sarcopenia had an estimated prevalence of 10.8% (sarcopenia and severe sarcopenia). The prevalence of sarcopenia was associated with advanced age, Caucasian race, reduced social support, illiteracy, low income, and the presence of comorbidities. Other studies that used the same approaches reported similar results 13,20. Nevertheless, when the cut-off points suggested by the EWGSOP were adopted, the prevalence of sarcopenia increased more than 7% of the actual value.

Reduced MM, strength, and functionality have been associated with adverse outcomes and mortality in patients with sarcopenia 21. The EWGSOP has proposed these factors as standards for detecting sarcopenia, and in the last 5 years, they have become the most commonly used criteria for diagnosing sarcopenia worldwide 11. Nevertheless, there are some issues associated with this operational definition. First, the wide variety of instruments used to identify MM, muscle strength and physical performance may produce differing values for these measures, which, in turn, may affect the reported prevalence of sarcopenia 8. Second, despite the growing evidence of their inadequacy 22 and the strong recommendation for the development of normative data for each population 11, the cut-off points for GS (0.8 m/s) and HS (20 kgf for women and 30 kgf for men) have been extensively used to classify populations outside of Northern Europe and North America, the regions in which these values were originally defined. Therefore, the reported sarcopenia prevalence rates may be inaccurate.

There are several examples of these issues mentioned above. Recently, in a systematic review on sarcopenia prevalence in Brazil, Diz et al. 23 found that of the 31 analyzed studies, 11 (35.5%) used all or some of the criteria recommended by the EWGSOP, and the overall prevalence of sarcopenia was 17%. Most of these studies used the cut-off points for HS and GS proposed by EWGSOP, and the estimated prevalence ranged from 4.1 to 65%. Consequently, one should be careful of uncritically accepting these results. There are many explanations for this huge variation in prevalence. Most explanations involve biases introduced during sample selection, the aforementioned use of different tools, and the cut-off values chosen to determine HS and GS. For example, the choice of cut-off points may explain the high sarcopenia prevalence rates of 15.4%, 13.9%, and 21.8% observed by Alexandre et al. 24, Barbosa-Silva et al. 25, and Martinez et al. 26, respectively, in their studies of community-dwelling and hospitalized older people in Brazil.

Some studies have proposed normative data for muscle strength and MM in the Brazilian population. Regarding the cut-off points for palmar grip strength, Budziareck et al. analyzed a convenience sample composed of 300 individuals from a city in the southern region of the country 27. The sample consisted of individuals with a mean age of 44.9 years (SD 18.5). In another study, Schlussel et al. presented the distribution of handgrip values by percentile but did not report some values that are usually reported, including the quartile and quintile values 28. Furthermore, the sample was composed of a small number of older people organized in only two age groups (60-69 years and ≥70 years).

Regarding the normative values for muscle mass, Barbosa-Silva et al. suggested cut-off values for MM in their study using DXA and calf circumference (CC) 25. The use of DEXA is standard. However, CC has a weak correlation with DXA, as shown in the systematic review published by Mijnarends et al., 29. For this reason, CC appears to be a poor proxy for MM.

The potential problems with cut-off values are not limited to Brazilian research. Studies from other Latin American and Asian countries have also adopted the cut-off points suggested by the EWGSOP screening algorithm 11. For example, Arango-Lopera et al. analyzed a community sample of 345 individuals over 70 years in Mexico City and found a prevalence of sarcopenia of 33.6% 30. Similarly, high sarcopenia prevalence rates were observed in the studies by Wu et al. 31 in Taiwan (12.8%) and by Yalcin et al. 32 in Turkey (29%). Indeed, it is important to reiterate that using the same cut-off values for populations with different genotypic and phenotypic characteristics may yield unreliable results, thus overestimating or underestimating the true prevalence 22.

Another strategy to define sarcopenia that is often used by the researchers is to accept the universality of the GS cut-off value of ≤0.8 m/s as a reference for slow walking. It was first proposed by Lauretani et al. and was recently proposed again by The Foundation of National Institute of Health Sarcopenia Project (FNIH) 33. The FNIH collected pooled data from multiple studies to develop criteria for weakness and low MM based on the association of both variables with slow GS 34. The FNIH found HS cut-off points of ≤16 kgf and 26 kgf and sarcopenia prevalence rates of 1.3% and 2.3% for women and men, respectively. Souza Barbosa et al. used the same strategy to estimate the prevalence of slow GS and weakness in a sample of elderly people from high- and middle-income countries 35. The maximum values of grip strength were 41.68 and 31.88 kgf in Canada and Brazil, respectively. The associated prevalences of weakness were 3.9 and 14.8%, respectively. This difference is probably a consequence of the cut-off points for GS that were used to determine a slow pace (GS ≤0.8 m/s) in these distinct populations. These results support Lourenço et al.'s report 22 that suggests that the clinically relevant reference value for GS in Latin America may be different from 0.8 m/s, and the use of this value to classify slow GS in older people may result in bias.

Additionally, Bahat et al. attempted to improve the general applicability of the sarcopenia criteria in Turkey by suggesting cut-off values for MM, HS, and CC 12. They studied two non-random samples composed of young and elderly adults and assessed MM using BIA, HS by its association with a slow pace(GS <0.8 m/s), and CC by identifying participants with MM <9.2 kg/m^2^ for men and <7.4 kg/m^2^ for women. They concluded that the cut-off thresholds for MM, HS, and CC were higher in Turkey than in other reference populations. However, their results may have been biased due to their acceptance of the universality of the GS cut-off value, the use of BIA as a reference for CC, and the unreliable use of CC as a measure of MM.

In addition, it is worth mentioning two other studies that suggest differences in HS reference values across different regions. Dodds et al. performed a meta-analysis highlighting the use of the British HS reference values in consensus definitions for sarcopenia across developed regions 36. However, they emphasized the need for different cut-off values in developing regions. Leong et al. analyzed the range of HS values in 125,462 healthy adults in 21 countries and highlighted the differences in HS among people from different geographic regions (Asia, Europe, Africa, and the Americas) 37. They strongly suggested that individual HS measurements should be interpreted using region/ethnic-specific reference ranges.

In addition to cut-off points for each population, other determinants of sarcopenia should be considered, including age, gender, geography, and individual risk factors 38. The methods used to define cut-off values may be directly influenced by these determinants. This is a concern for HS classification using BMI, which was the strategy used in the present study. Notwithstanding the fact that Budzireck et al. 27 did not find a correlation between BMI and HS, there have been several studies supporting this approach 15.

Although they used cut-off values from another population, Alexandre et al. 24 identified in a Brazilian population that age and income were also risk factors for sarcopenia, as observed in the present study. The first is well established in the literature 38. However, income and other sociodemographic variables need to be further studied, especially in developing countries.

There were some limitations in the present study. The use of anthropometric equations capable of predicting MM are practical and simple methods that are convenient in low income countries 11,39. However, the accuracy of this method is not ideal. Another issue is that the formula used for the estimation of MM in the present study calculated the total skeletal MM. Some investigators suggest that appendicular MM should be used 8. Therefore, there is a possibility that the sarcopenia prevalence determined in the present study was an underestimate.

The sample in the present study was obtained from a health care provider, with different sociodemographic data, income levels and educational levels, and the results should not be generalized. On the other hand, 25% of those using the Brazilian health system are supported by the Supplementary Health Care System (a total of 50 million individuals and 7 million over 60 years); therefore, it is very important to know the health status of this population subgroup 40.

## CONCLUSION

Previous studies have indicated that the prevalence of sarcopenia varies greatly depending on the MM, HS and GS cut-off values employed to estimate it. In the present study, the prevalence rates of sarcopenia and its associated factors were similar to those reported in other studies once the MM, HS and GS values were tailored for the specific populations and adopted as the diagnostic criteria.

## AUTHOR CONTRIBUTIONS

All authors participated in the preparation of this manuscript, data collection, analysis of the information and discussion.

## Figures and Tables

**Table 1:** Sociodemographic characteristics, health statuses and comorbidities in community-dwelling individuals aged 65 years or older who are residents of the northern region of Rio de Janeiro, Brazil, 2010. Fibra-RJ study. (n=745)

		No sarcopenia	Pre-sarcopenia	Sarcopenia	Total
		n (%)	n (%)	n (%)	n (%)
**Sex**	Female	423 (70.5)	39 (60)	62 (77.5)	524 (70.3)
	Male	177 (29.5)	26 (40)	18 (22.5)	221 (29.7)
**Race**	Other	245 (40.8)	16 (24.6)	23 (28.8)	284 (38.1)
	Caucasian	355 (59.2)	49 (75.4)	57 (71.3)	461 (61.9)
**Age (years)**	65 - 74	304 (50.7)	11 (16.9)	5 (6.3)	320 (43.0)
	75 - 84	245 (40.8)	45 (69.2)	39 (48.8)	329 (44.2)
	>85	51 (8.5)	9 (13.8)	36 (45)	96 (12.9)
**Marital status**	Married/living with partner	278 (46.3)	25 (38.5)	16 (20)	319 (42.8)
	Divorced/separated	49 (8.2)	3 (4.6)	6 (7.5)	58 (7.8)
	Single	58 (9.7)	11 (16.9)	13 (16.3)	82 (11)
	Widower	215 (35.8)	26 (40)	45 (56.3)	286 (38.4)
**Educational level (years)**	Illiterate	13 (2.2)	1 (1.5)	3 (3.8)	17 (2.3)
	1 - 5	139 (23.2)	15 (23.1)	26 (32.5)	180 (24.2)
	6 - 11	228 (38)	22 (33.8)	32 (40.0)	282 (37.9)
	>12	220 (36.7)	27 (41.5)	19 (23.8)	266 (35.7)
**Income (MW**^a^**)**	0 - 2	88 (15.4)	9 (14.1)	23 (30.3)	120 (16.9)
	2.1 - 5	198 (34.7)	22 (34.4)	27 (35.5)	247 (34.7)
	>5. 1	285 (49.9)	33 (51.6)	26 (35.2)	344 (48.4)
**Comorbidities**	0 - 1	248 (41.3)	35 (53.8)	18 (22.5)	301 (40.4)
	2 - 3	286 (47.7)	26 (40.0)	48 (60.0)	360 (48.3)
	>4	66 (11.0)	4 (6.2)	14 (17.5)	84 (11.3)
**IADL**^b^	Independent	298 (49.7)	35 (53.8)	19 (23.8)	352 (47.2)
	Dependent	302 (50.3)	30 (46.2)	61 (76.3)	393 (52.8)
**BADL**^c^	Independent	490 (81.7)	56 (86.2)	61 (76.3)	607 (81.5)
	Dependent	110 (18.3)	9 (13.8)	19 (23.8)	138 (18.5)
**Health self-perception**	Very Bad - Bad	30 (5.0)	0 (0.0)	6 (7.5)	36 (4.8)
	Normal	234 (39.0)	24 (36.9)	32 (40.0)	290 (38.9)
	Good - Very Good	336 (56.0)	41 (63.1)	42 (52.5)	419 (56.2)
**Falls**	0	432 (72.0)	51 (78.5)	52 (65.0)	535 (71.8)
	1 - 2	141 (23.5)	12 (18.5)	21 (26.3)	174 (23.4)
	≥3	27 (4.5)	2 (3.1)	7 (8.8)	36 (4.8)
**Total**		600 (80.5)	65 (8.7)	80 (10.7)	745 (100)

a Minimum wage (2009-2010: U$ 265); there were 34 missing data points;

b Instrumental activities of daily living;

c Basic activities of daily living

**Table 2:** Sample tailored cut-off values for handgrip strength, gait speed and muscle mass among community-dwelling individuals aged 65 years or older who are residents of the northern region of Rio de Janeiro, Brazil, 2010. (n=745).

	Cut-off values by gender			
	Men		Women	
	BMI^a^	Handgrip strength (kgf)	BMI	Handgrip strength (kgf)
**Muscle strength**^b^	≤22.40	16.8	≤24.12	13.3
	22.40< to ≤25.51	23.3	24.12< to ≤26.92	14.0
	25.51< to ≤28.33	23.3	26.92< to ≤30.26	14.0
	>28. 33	23.4	>30.26	14.7

	Height (m)	Gait speed (m/s)	Height (m)	Gait speed (m/s)
**Physical performance**^c^	≤1.68	<0.65	≤1.54	<0.60
	>1.68	<0.73	>1.54	<0.69
**Muscle mass**^d^	8.83		6.64	

a BMI - Body Mass Index;

b Muscle strength estimated through handgrip strength using the Body Mass Index quartile and the sex-specific lowest quintile;

c Physical performance estimated through gait speed using the usual pace for 4.6 meters, median height and sex-specific lowest quintile;

d Muscle mass estimated through anthropometric measurement with sex-specific lowest quintile (kg/m^2^).

**Table 3:** Sarcopenia prevalence estimated by the tailored and EWGSOP cutoff points among community-dwelling individuals aged 65 years or older who are residents of the northern region of Rio de Janeiro, Brazil, 2010, stratified by sex. (n=745)

		Male (n %)	Female (n %)	Total (n %)
Fibra-RJ	Normal	177 (80.1)	421 (80.7)	598 (80.5)
	Pre-sarcopenia	26 (11.8)	39 (7.5)	65 (8.7)
	Sarcopenia	12 (5.4)	39 (7.5)	51 (6.9)
	Severe sarcopenia	6 (2.7)	23 (4.4)	29 (3.9)
EWGSOP	Normal	177 (80.1)	421 (80.7)	598 (80.5)
	Pre-sarcopenia	9 (4.1)	2 (0.4)	11 (1.5)
	Sarcopenia	20 (9.0)	42 (8.0)	62 (8.3)
	Severe sarcopenia	15 (6.8)	57 (10.9)	72 (9.7)

*p*<0.001

**Table 4:** Sarcopenia and associated factors among individuals aged 65 years or older who are residents of the northern region of Rio de Janeiro, Brazil, 2010. (N=680^a^)

Independent variables	Categories	Univariate		Multivariate	
		OR (95% CI)	*p*-value	OR (95% CI)	*p*-value
Sex	Male	1		1	
	Female	1.4 (0.8-2.50)	0.19	0.63 (0.29-1.36)	0.24
Race	Other	1		1	1
	Caucasian	1.7 (1.02-2.85)	0.03	1.89 (1.02-3.52)	0.04
Age group (years)	65-74	1		1	1
	75-84	9.67 (3.75-24.92)	<0.001	9.72 (3.62-26.12)	0.000
	>85	42.9 (16.0-114.5)	<0.001	37.2 (12.35-112.48)	0.000
Marital status	Married/living with partner	1		1	
	Divorced/separated	2.1 (0.74-5.70)	0.09	1.98 (0.58-6.72)	0.27
	Single	3.89 (1.70-8.50)	<0.001	6 (2.20-16.39)	0.000
	Widower	3.63 (2.0-6.61)	<0.001	2.49 (1.15-5.37)	0.02
Educational level (years)	>12	1		1	
	Illiterate	2.67 (0.70-10.20)	0.15	0.48 (0.09-2.60)	0.4
	1-5	2.16 (1.15-4.0)	0.01	0.45 (1.18-1.12)	0.08
	6-11	1.62 (0.89-2.95)	0.09	1.39 (0.65-2.95)	0.39
Income (MW^b^)	>5. 1	1		1	
	0-2	2.86 (0.21-0.62)	<0.001	3.64 (1.58-8.39)	0.002
	2. 1-5	1.94 (0.84-2.63)	0.16	2.44 (1.20-4.94)	0.013
Comorbidities	0-1	1		1	
	2-3	2.31 (1.31-4)	0.004	2.55 (1.28-5.08)	0.008
	>4	2.9 (1.38-6.1)	0.005	3.26 (1.28-8.30)	0.013
IADL^d^	Independent	1		1	
	Dependent	3.1 (1.84-5.43)	<0.001	1.64 (0.86-3.11)	0.13
BADL^d^	Independent				
	Dependent	1.38 (0.79-2.41)	0.24	-	-
Health self-perception	Good-Very Good				
	Very Bad-Bad	1.6 (0.62-4.0)	0.32	-	-
	Regular	1 (0.67-1.78)	0.70	-	-
Falls	0	1			
	1-2	1.23 (0.72-2.10)	0.44	-	-
	≥3	2.1 (0.89-5.10)	0.87	-	-
Caloric expenditure^e^	First quintile	1.1 (0.65-2.0)	0.60	-	-

a 65 individuals (pre-sarcopenic) were excluded;

b Minimum wage (2009-2010: U$ 265);

c Instrumental activities of daily living;

d Basic activities of daily living.);

e Caloric expenditure calculated by Minnesota Leisure Time Activities.

Hosmer-Lemeshow (0.84)

## References

[1] IBGE (2015). Síntese de indicadores sociais: uma análise das condições de vida da população brasileirea.

[2] Schmidt MI, Duncan BB, Azevedo e Silva G, Menezes AM, Monteiro CA, Barreto SM (2011). Chronic non-communicable diseases in Brazil: burden and current challenges. Lancet.

[3] Kraemer RR, Castracane VD (2015). Novel insights regarding mechanisms for treatment of sarcopenia. Metabolism.

[4] Cederholm T, Morley JE (2015). Sarcopenia: the new definitions. Curr Opin Clin Nutr Metab Care.

[5] Visser M, Goodpaster BH, Kritchevsky SB, Newman AB, Nevitt M, Rubin SM (2005). Muscle mass, muscle strength, and muscle fat infiltration as predictors of incident mobility limitations in well-functioning older persons. J Gerontol A Biol Sci Med Sci.

[6] Tarantino U, Piccirilli E, Fantini M, Baldi J, Gasbarra E, Bei R (2015). Sarcopenia and fragility fractures: molecular and clinical evidence of the bone-muscle interaction. J Bone Joint Surg Am.

[7] Tarantino U, Baldi J, Celi M, Rao C, Liuni FM, Iundusi R (2013). Osteoporosis and sarcopenia: the connections. Aging Clin Exp Res.

[8] Baumgartner RN, Koehler KM, Gallagher D, Romero L, Heymsfield SB, Ross RR (1998). Epidemiology of sarcopenia among the elderly in New Mexico. Am J Epidemiol.

[9] Janssen I, Baumgartner RN, Ross R, Rosenberg IH, Roubenoff R (2004). Skeletal muscle cutpoints associated with elevated physical disability risk in older men and women. Am J Epidemiol.

[10] Keevil VL, Romero-Ortuno R (2015). Ageing well: a review of sarcopenia and frailty. Proc Nutr Soc.

[11] Cruz-Jentoft AJ, Baeyens JP, Bauer JM, Boirie Y, Cederholm T, Landi F (2010). Sarcopenia: European consensus on definition and diagnosis: Report of the European Working Group on Sarcopenia in Older People. Age Ageing.

[12] Bahat G, Tufan A, Tufan F, Kilic C, Akpinar TS, Kose M (2016). Cut-off points to identify sarcopenia according to European Working Group on Sarcopenia in Older People (EWGSOP) definition. Clin Nutr.

[13] Reijnierse EM, Trappenburg MC, Leter MJ, Blauw GJ, Sipila S, Sillanpaa E (2015). The Impact of Different Diagnostic Criteria on the Prevalence of Sarcopenia in Healthy Elderly Participants and Geriatric Outpatients. Gerontology.

[14] Lourenco RA, Sanchez MA, Moreira VG, Ribeiro PCC, Perez M, Campos GC (2015). Frailty in Older Brazilians - FIBRA-RJ: research methodology on frailty, cognitive disorders and sarcopenia. Revista do Hospital Pedro Ernesto.

[15] Fried LP, Tangen CM, Walston J, Newman AB, Hirsch C, Gottdiener J (2001). Frailty in older adults: evidence for a phenotype. J Gerontol A Biol Sci Med Sci.

[16] Lee RC, Wang Z, Heo M, Ross R, Janssen I, Heymsfield SB (2000). Total-body skeletal muscle mass: development and cross-validation of anthropometric prediction models. Am J Clin Nutr.

[17] Newman AB, Kupelian V, Visser M, Simonsick E, Goodpaster B, Nevitt M (2003). Sarcopenia: alternative definitions and associations with lower extremity function. J Am Geriatr Soc.

[18] Delmonico MJ, Harris TB, Lee JS, Visser M, Nevitt M, Kritchevsky SB (2007). Alternative definitions of sarcopenia, lower extremity performance, and functional impairment with aging in older men and women. J Am Geriatr Soc.

[19] Moreira VG, Nascimento JS, Lourenço RA (2017). Prevalence of Sarcopenia and its associated factors: the impact of different cutoff values. Innovation in Aging.

[20] Wen X, An P, Chen WC, Lv Y, Fu Q (2015). Comparisons of sarcopenia prevalence based on different diagnostic criteria in Chinese older adults. J Nutr Health Aging.

[21] Perez-Zepeda MU, Sgaravatti A, Dent E (2017). Sarcopenia and post-hospital outcomes in older adults: A longitudinal study. Arch Gerontol Geriatr.

[22] Lourenco RA, Pérez-Zepeda M, Gutiérrez-Robledo L, García-García FJ, Rodríguez Maãas L (2015). Performance of the European Working Group on Sarcopenia in Older People algorithm in screening older adults for muscle mass assessment. Age Ageing.

[23] Diz JB, Leopoldino AA, Moreira BS, Henschke N, Dias RC, Pereira LS (2017). Prevalence of sarcopenia in older Brazilians: A systematic review and meta-analysis. Geriatr Gerontol Int.

[24] Alexandre Tda S, Duarte YA, Santos JL, Wong R, Lebrão ML (2014). Prevalence and associated factors of sarcopenia among elderly in Brazil: findings from the SABE study. J Nutr Health Aging.

[25] Barbosa-Silva TG, Bielemann RM, Gonzalez MC, Menezes AM (2016). Prevalence of sarcopenia among community-dwelling elderly of a medium-sized South American city: results of the COMO VAI? study. J Cachexia Sarcopenia Muscle.

[26] Martinez BP, Batista AK, Gomes IB, Olivieri FM, Camelier FW, Camelier AA (2015). Frequency of sarcopenia and associated factors among hospitalized elderly patients. BMC Musculoskelet Disord.

[27] Budziareck MB, Pureza Duarte RR, Barbosa-Silva MC (2008). Reference values and determinants for handgrip strength in healthy subjects. Clin Nutr.

[28] Schlussel MM, dos Anjos LA, de Vasconcellos MT, Kac G (2008). Reference values of handgrip dynamometry of healthy adults: a population-based study. Clin Nutr.

[29] Mijnarends DM, Meijers JM, Halfens RJ, ter Borg S, Luiking YC, Verlaan S (2013). Validity and reliability of tools to measure muscle mass, strength, and physical performance in community-dwelling older people: a systematic review. J Am Med Dir Assoc.

[30] Arango-Lopera VE, Arroyo P, Gutiérrez-Robledo LM, Pérez-Zepeda MU (2012). Prevalence of sarcopenia in Mexico City. European geriatric medicine.

[31] Wu IC, Lin CC, Hsiung CA, Wang CY, Wu CH, Chan DC (2014). Epidemiology of sarcopenia among community-dwelling older adults in Taiwan: a pooled analysis for a broader adoption of sarcopenia assessments. Geriatr Gerontol Int.

[32] Yalcin A, Aras S, Atmis V, Cengiz OK, Varli M, Cinar E (2016). Sarcopenia prevalence and factors associated with sarcopenia in older people living in a nursing home in Ankara Turkey. Geriatr Gerontol Int.

[33] Lauretani F, Russo CR, Bandinelli S, Bartali B, Cavazzini C, Di Iorio A (2003). Age-associated changes in skeletal muscles and their effect on mobility: an operational diagnosis of sarcopenia. J Appl Physiol.

[34] Alley DE, Shardell MD, Peters KW, McLean RR, Dam TT, Kenny AM (2014). Grip strength cutpoints for the identification of clinically relevant weakness. J Gerontol A Biol Sci Med Sci.

[35] de Souza Barbosa JF, Zepeda MU, Beland F, Guralnik JM, Zunzunegui MV, Guerra RO (2016). Clinically relevant weakness in diverse populations of older adults participating in the International Mobility in Aging Study. Age (Dordr).

[36] Dodds RM, Syddall HE, Cooper R, Kuh D, Cooper C, Sayer AA (2016). Global variation in grip strength: a systematic review and meta-analysis of normative data. Age Ageing.

[37] Leong DP, Teo KK, Rangarajan S, Kutty VR, Lanas F, Hui C (2016). Reference ranges of handgrip strength from 125,462 healthy adults in 21 countries: a prospective urban rural epidemiologic (PURE) study. J Cachexia Sarcopenia Muscle.

[38] Shaw SC, Dennison EM, Cooper C (2017). Epidemiology of Sarcopenia: Determinants Throughout the Lifecourse. Calcif Tissue Int.

[39] Rech CR, Dellagrana RA, Marucci MdFN, Petroski EL (2012). Validity of anthropometric equations for the estimation of muscle mass in the elderly. Revista Brasileira de Cineantropometria & Desempenho Humano.

[40] Oliveira M AD, Veras R, Gomes G, Estrella K, Neves R, Assalim V (2016). Idosos na saúde suplementar: uma urgência para a saúde da sociedade e sustentabilidade do setor.

